# Effectiveness of Amlodipine in the Control of 24-Hour Blood Pressure in Mild-to-Moderate Essential Hypertension: A Prospective, Multicenter, Observational Real-World Study from India

**DOI:** 10.7759/cureus.38272

**Published:** 2023-04-28

**Authors:** Prabhakar D, Santanu Guha, Rahul Rathod, Kumar Gaurav

**Affiliations:** 1 Cardiology, Ashwin Cardiology Clinic, Chennai, IND; 2 Cardiology, Nightingle Hospital, Kolkata, IND; 3 Family Medicine, Dr. Reddy’s Laboratories Ltd, Hyderabad, IND; 4 Pharmacology, Dr. Reddy’s Laboratories Ltd, Hyderabad, IND

**Keywords:** diastolic blood pressure, systolic blood pressure, abpm, hypertension, amlodipine

## Abstract

Background

Calcium-channel blockers (CCB) are a mainstay in the management of hypertension (HTN), and amlodipine is the preferred CCB due to its longer half-life and better safety.

Patients and methods

This practice-based, observational, real-world evidence study assessed the 24-hour ambulatory blood pressure (ABP) control in mild-to-moderate essential hypertensive patients (treatment naïve receiving amlodipine monotherapy, or as add-on therapy) in inadequately controlled blood pressure (BP goal not met after one month of existing therapy). One hundred four (62M/42F) patients between 18 and 65 years of age who received amlodipine 5/10 mg/day for >8 weeks were included after obtaining informed written consent. The primary outcome was a change from baseline in the mean 24-h SBP and DBP on ABP monitoring at eight (±1) weeks. Secondary outcomes were different from baseline in the mean morning, day-time, night-time, and last six-hour dosing interval period. Other outcomes assessed BP variability (dippers and non-dippers), and responder rates based on ABP monitoring and in-clinic trough cuff measurements. Safety outcomes included adverse effects reported, electrocardiogram, and laboratory abnormalities (hepatic and renal function). Changes in BP parameters in different sub-groups (age, gender, BMI, physical activity, occupation, duration of hypertension, the total daily dose of amlodipine, type of amlodipine therapy, and hypertension severity at baseline) were estimated.

Results

Of the 104 patients, 49 patients (completed eight weeks) were included in the per-protocol (PP) data for efficacy analysis. Safety analysis was done on the intent-to-treat (ITT) dataset. Significant reductions (p<0.0001) from baseline in all BP measurements on ABPM were observed at the end of eight weeks. Similar improvements were observed in office BP recordings. There was a marginal but non-significant (p>0.05) increase in the proportion of dippers and extreme dippers with amlodipine at the end of the eight-week treatment period.

Conclusions

Amlodipine 5/10 mg/day therapy used as monotherapy or adjuvant therapy provided significant reductions in both office BP and ambulatory BP over eight weeks.

## Introduction

Hypertension (HTN) was ranked as the third most important risk factor for the attributable burden of disease in South Asia in 2010 [1]. Estimates by the WHO (2008) put the prevalence of Hypertension at 33.2% in men and 31.7% in women with an overall prevalence of 32.5% [2]. In the “Heart Disease and Stroke Statistics 2021 Update,” the age-adjusted US prevalence of hypertension between 2015 and 2018 in those over the age of 20 years was 51.7% in males and 42.8% in females [3]. Worldwide data from 2005 for the global burden of hypertension project a prevalence of 22.9% for men and 23.6% for women by 2025 in India [4]. Hypertension contributes to macrovascular and microvascular complications leading to stroke, myocardial infarction, heart failure, renal failure, etc. [5]. Persistent and round-the-clock blood pressure (BP) control is essential to prevent complications [5].

The use of 24-hour ambulatory BP monitoring (ABPM) greatly improves the ability to assess the time course of antihypertensive treatment-associated lowering of BP throughout the dosing interval [6]. However, ABPM is not used routinely in the management of hypertensive patients because of its prohibitive cost. Ambulatory monitoring studies have documented reproducible 24-h diurnal variations in BP, characterized by a period of low values during sleep, an early-morning increase in pressures, and a plateau period while the individual is awake and active. Hypertensive patients who display the typical nocturnal decrease in BP are termed “dippers,” whereas patients in whom the nocturnal decrease in BP is absent or blunted are termed “non-dippers” [7].

Amlodipine, a third-generation dihydropyridine (DHP) calcium channel blocker, is a long-acting, lipophilic drug that causes vasodilation of vascular smooth muscle cells resulting in decreased peripheral vascular resistance (PVR). Due to its high oral bioavailability (60%-80%) and long half-life (35-50 hours), it is administered as once-daily therapy, which is favorable for patient compliance. Although amlodipine is one of the preferred first-line anti-hypertensive, data on 24-hour BP control is sparsely reported [8]. This study collected real-world data from the Indian population on 24-hour ambulatory BP (ABP) control in patients with mild-to-moderate essential hypertension who were treated with amlodipine-based antihypertensive therapy.

## Materials and methods

Study design and setting

This quasi-experimental study was a prospective, real-world, observational study that assessed of effectiveness and safety of oral amlodipine 5/10 mg daily for eight (±1) weeks for treatment of mild-to-moderate essential hypertension at two cardiac care centers from different regions of India.

Informed consent and ethics

The study was conducted in accordance with the principles of the Declaration of Helsinki (World Medical Association) and Good Clinical Practice (GCP) guidelines issued by the ICMR and CDSCO, Govt. of India. Informed consent was obtained from all patients, and strict patient confidentiality was maintained. The study was approved by the respective Institutional Ethics Committees of the study sites and was registered with the clinical trials registry of India (CTRI/2020/08/027402).

Study participants

A total of 104 patients (62 male, 42 female) having mild-to-moderate essential hypertension (>140 mmHg SBP and/or >90 mmHg DBP) and who were prescribed oral amlodipine 5/10 mg/day as monotherapy or add-on to prior antihypertensive therapy were included. Patients of either gender between 18 and 65 years of age, who were either treatment naïve or inadequately controlled with other hypertensive drugs (i.e., BP goal not met after one month of antihypertensive therapy) were included.

Non-ambulatory patients and those with secondary hypertension, receiving any other calcium-channel blockers (CCBs), known hypersensitivity to amlodipine, and known hepatic or renal impairment were excluded. Pre-menopausal women not practicing any birth control measures, pregnant, or those who were breastfeeding were excluded. Patients with New York Heart Association (NYHA) functional class III or IV congestive heart failure (CHF), unstable angina, acute myocardial infarction, heart surgery, or stroke within the previous six months were excluded. Those with any type of cardiac arrhythmia, or with any valvular heart disease with hemodynamic repercussion, receiving chronic administration of oral anticoagulants or digoxin, or having severe, uncontrolled hypertension or any form of secondary hypertension were not included. Patients with any other clinical conditions which, in the opinion of the investigator, would not allow for the safe completion of the protocol were excluded.

Study procedure

Data were collected and recorded in a study-specific electronic data capture tool. Patient information about their clinical condition, comorbidities, complications, and details of other treatments received by them was collected. This being an observational study, there were no study-specific treatments, and all treatments for the patients were based on the discretion of the treating clinician. Only those patients who were prescribed amlodipine at the treating clinician’s discretion and completed eight (±1) weeks of therapy with amlodipine 5/10 mg per day were included in the final analysis.

Ambulatory BP monitoring

In the clinic, BP was measured using a standard mercury/aneroid/digital oscillometric sphygmomanometer in accordance with standard guidelines. Measures were taken three times in the seated position, at intervals of 2 min, and the mean value was used [4]. Eligible participants of the study were then provided ABPM devices, which were worn for 24 hours. Participants returned the next day after the completion of 24 hours of the ABPM device. These assessments were made at baseline and then after 8 (±1) weeks. The schedule of BP measurement during this 24-h period was every 20 minutes during the day (0800 to 2200 h) and every 60 min during the night (2200 to 0800 h). Recordings with more than 70% measurements and only those with at least one reading per hour during the night and early morning hours were considered valid and included for analysis. From the ABPM recordings, the mean values of SBP and DBP were calculated for the morning (0600-1000 h), day-time (0800-2200 h), and night-time (2200-0800 h).

Laboratory assessments

No study-specific laboratory investigations were planned. Laboratory investigations or ECG, if any were performed at the discretion of the investigator based on his/her clinical judgment and in the interest of patient safety. Any such laboratory data were recorded and evaluated for any significant changes.

Study outcomes

The primary outcome was change from baseline in the mean 24-h SBP and DBP on ABPM at the end of study period (8 ± 1 weeks). Secondary outcomes were change from baseline in SBP and DBP values for morning, day-time, night-time, and last six-hour dosing interval period (relative to dose time) at the end of study. Other outcomes were change from baseline in office (sitting SBP/DBP measured by manual cuff sphygmomanometer) BP measurements. Responder rates were assessed based on measurements from ABPM and in-clinic trough cuff measurements. BP variability was evaluated using proportion of dippers (10%-20% reduction in night-time SBP relative to daytime) and non-dippers. Safety outcomes included adverse effects reported by the patients, and any abnormal findings reported through routine investigations. All assessments were made at baseline and at eight (±1) weeks.

Statistical methods

The sample size was calculated based on the primary endpoint of the study, i.e., change from baseline in the mean 24-hr SBP and DBP on ABPM at the end of eight weeks of amlodipine-based therapies. Literature reports a reduction in SBP with amlodipine monotherapy and add-on therapy at the end of eight (±1) weeks is 7.6 (8.3) and 13.1 (10.2) mmHg, respectively, and the reduction in DBP is 5.3 (5.8) and 9.3 (7.7) mmHg, respectively [9]. Using a one-sample mean test (two-sided) to detect a reduction in SBP (H1) by 13.1 (10.2) mmHg with amlodipine combination therapy versus a reduction in SBP (H0) by 7.6 (8.3) mmHg with amlodipine monotherapy at the end of eight (±1) weeks, the sample size needed was 37 at 90% power and alpha 0.05 (95% confidence). However, we planned to include 150 patients in the study anticipating stratification based on gender and hypertension severity.

The effect of amlodipine therapy on BP was estimated as differences in the BP measurements at baseline and at eight weeks using repeat measures analysis of covariance (ANCOVA), with age, gender, BMI, physical activity, occupation, duration of hypertension, a total daily dose of amlodipine, type of amlodipine therapy (monotherapy/combination therapy), and hypertension severity at baseline as covariates. A multivariate analysis was performed with BP parameters as dependent variables, and sub-groups as independent variables.

McNemar’s test was used to compare the dippers and non-dippers at baseline and end of study. Efficacy analysis was done on the per-protocol (PP) dataset which included 49 patients who completed the study as per the protocol. Safety analysis was done on the intent-to-treat (ITT) dataset of all 104 patients who received at least one dose of amlodipine therapy. All analyses were done with Stata-IC 13 for Windows (StataCorp LLC, TX, USA) using two-sided tests with alpha 0.05.

## Results

Demography and baseline data

Data from 104 patients were collected and evaluated (ITT dataset). Only 49 patients completed the study and were included in the PP dataset. Reasons for dropout could not be ascertained and were due to follow-up loss. Table 1 presents the demographic data and baseline vital parameters in ITT and PP datasets.

**Table 1:** Demographic data and vital parameters at baseline in PP (n=49) and ITT (n=104) datasets BMI: Body mass index; DBP: Diastolic blood pressure; PP: Per protocol; SBP: Systolic blood pressure; SD: Standard deviation

	Mean	Median	SD	95% CI of mean
PP dataset (n=49)				
Age (yrs.)	53.33	56.00	9.35	50.6 to 56.0
BMI (kg/sq.m.)	29.59	29.41	4.11	28.4 to 30.8
Duration of hypertension (yrs.)	5.50	4.00	5.78	3.8 to 7.2
SBP (mmHg)	153.69	152.00	11.35	150.4 to 157.0
DBP (mmHg)	88.37	90.00	10.03	85.5 to 91.2
Pulse rate (per min.)	80.80	81.00	12.11	77.3 to 84.3
Body Temperature (^0^C)	97.81	97.90	0.60	97.6 to 98.0
Respiratory rate (per min.)	17.97	18.00	0.45	17.8 to 18.1
ITT dataset (n=104)				
Age (yrs.)	53.29	56.00	9.50	51.4 to 55.1
BMI (kg/sq.m.)	29.11	28.97	4.53	28.2 to 30.0
Duration of hypertension (yrs.)	6.57	4.50	6.78	5.3 to 7.9
SBP (mmHg)	154.38	152.00	12.27	152.0 to 156.8
DBP (mmHg)	90.06	90.00	9.85	88.1 to 92.0
Pulse rate (per min.)	80.96	81.00	12.09	78.6 to 83.3
Body Temperature (^0^C)	97.65	97.80	0.67	97.5 to 97.8
Respiratory rate (per min.)	18.12	18.00	0.51	18.0 to 18.2

Table 2 presents the profile of patients in the ITT and PP dataset. Rosuvastatin, atorvastatin, metoprolol, and telmisartan were the most used drugs in the patients prior to enrolment, whereas metoprolol, rosuvastatin and telmisartan were the most co-prescribed concomitant drug therapy. About 25% patients received additional drugs for hypertension.

**Table 2:** Profile of patients in PP (n=49) and ITT (n=104) datasets GIT: Gastrointestinal system; H/O: History of; ITT: Intend-to-treat; PP: Per protocol; N/A: Not available; Stage-1: Systolic blood pressure 140-149 mmHg, or diastolic blood pressure 90-99 mmHg; Stage-2: Systolic blood pressure 160-179 mmHg, or diastolic blood pressure 100-109 mmHg; WHO: World health Organization

		PP dataset (n=49)	ITT dataset (n=104)
		No. (%)	No. (%)
Occupation	Service	12 (24.5%)	31 (29.8%)
	Self-employed / business	10 (20.4%)	18 (17.3%)
	Unemployed	18 (36.7%)	38 (36.5%)
	Retired	9 (18.4%)	17 (16.3%)
Physical activity in life	Sedentary	23 (46.9%)	46 (44.2%)
	Mild-to-moderate activity	13 (26.5%)	37 (35.6%)
	Heavy activity	13 (26.5%)	21 (20.2%)
Gender	Male	30 (61.2%)	62 (59.6%)
	Female	19 (38.8%)	42 (40.4%)
Diet	Vegetarian	15 (30.6%)	21 (20.2%)
	Non-vegetarian (Mixed)	34 (69.4%)	83 (79.8%)
Smoking	Never smoked	39 (79.6%)	80 (76.9%)
	Former smoker	6 (12.2%)	13 (12.5%)
	Current smoker	4 (8.2%)	11 (10.6%)
Duration of smoking	≤9 years	7 (70.0%)	13 (54.2%)
	10-19 years	1 (10.0%)	7 (29.2%)
	≥20 years	2 (20.0%)	4 (16.7%)
Alcohol	Never	38 (77.6%)	79 (76.0%)
	Former	4 (8.2%)	5 (4.8%)
	Current	7 (14.3%)	20 (19.2%)
Family history of hypertension	Present	33 (67.3%)	74 (71.2%)
	Absent	16 (32.7%)	30 (28.8%)
Severity of hypertension	Stage-1	34 (69.4%)	60 (57.7%)
	Stage-2	15 (30.6%)	44 (42.3%)
Amlodipine daily dose	Amlodipine 5 mg/day	28 (57.1%)	68 (65.4%)
	Amlodipine 10 mg/day	21 (42.9%)	36 (34.6%)
Duration of hypertension	<5 years	25 (51.0%)	52 (50.0%)
	≥5 years	24 (49.0%)	52 (50.0%)
BMI categories (WHO criteria)	Underweight (<18.5 kg/sq.m.)	0 (0.0%)	1 (1.0%)
	Normal (18.5 to <25.0 kg/sq.m.)	6 (12.2%)	18 (17.3%)
	Overweight (25.0 to <30.0 kg/sq.m.)	21 (42.9%)	41 (39.4%)
	Obese (≥30.0 kg/sq.m.)	22 (44.9%)	44 (42.3%)
Age group	<50 years	18 (36.7%)	32 (30.8%)
	≥50 years	31 (63.3%)	72 (69.2%)
Duration of therapy	8-9 wks.	44 (89.8%)	44 (42.3%)
	16-24 wks.	5 (10.2%)	5 (4.8%)
	N/A	0 (0.0%)	55 (52.9%)
Systemic abnormality	General examination	1 (2.0%)	1 (1.0%)
	Cardiovascular	7 (14.3%)	39 (37.5%)
	Respiratory	2 (4.1%)	3 (2.9%)
	Abdominal/GIT	1 (2.0%)	5 (4.8%)
Amlodipine therapy	Monotherapy	17 (34.7%)	35 (33.6%)
	Combination therapy	32 (65.3%)	69 (66.4%)

Reduction in SBP and DBP

The mean 24-hr. reduction was (-)10.82 and (-)6.25 mmHg with 5 mg amlodipine and (-)19.81 and (-)11.19 mmHg with 10 mg amlodipine for SBP and DBP, respectively (p<0.0001). The mean morning, daytime, night-time, and last six-hour reductions in SBP were (-)14.06, (-)18.63, (-)20.42 and (-)13.51, respectively (p<0.0001), and DBP reductions were (-)7.78, (-)10.27, (-)11.07, and (-)6.44, respectively (p<0.0001). Table 3 presents ambulatory values of SBP and DBP, respectively, for baseline, end of study and change from baseline (adjusted) in PP dataset (n=49).

**Table 3:** 24-hr. ambulatory BP (mm Hg) at baseline and EoS (8 wks.) in PP dataset (n=49) * Change in BP adjusted (ANCOVA) for age, gender, BMI, duration of hypertension, occupation, type of amlodipine therapy, hypertension severity, and physical activity. BP: Blood pressure; DBP: Diastolic blood pressure; PP: Per protocol; SBP: Systolic blood pressure; SD: Standard deviation

		Baseline	8 weeks	Change (adjusted)*	t-test	Effect size
	N	Mean (SD)	Mean (SD)	Mean (SD)	P	Cohen’s d
SBP (mm Hg)						
24-hr BP	49	139.31 (13.07)	124.63 (9.51)	-13.00 (12.44)	<0.0001	1.12
Morning BP	49	137.82 (17.03)	124.31 (12.10)	-7.00 (7.55)	<0.0001	0.83
Day-time BP	49	141.33 (13.90)	127.27 (9.64)	-14.00 (12.98)	<0.0001	1.34
Night-time BP	49	132.27 (14.72)	113.63 (11.83)	-7.00 (7.84)	<0.0001	1.39
Last 6-hr dosing interval BP	49	140.98 (09.32)	120.57 (11.06)	-18.00 (13.70)	<0.0001	1.45
DBP (mm Hg)						
24-hr BP	49	82.18 (11.88)	73.82 (8.51)	-9.00 (8.57)	<0.0001	0.70
Morning BP	49	82.18 (14.07)	75.74 (10.41)	-20.30 (15.32)	<0.0001	0.55
Day-time BP	49	83.49 (12.62)	75.71 (9.18)	-9.75 (11.00)	<0.0001	0.81
Night-time BP	49	76.49 (10.69)	66.22 (8.02)	-13.20 (16.30)	<0.0001	1.04
Last 6-hr dosing interval BP	49	81.93 (09.61)	72.61 (7.79)	-6.60 (10.75)	<0.0001	0.67

**Figure 1:**
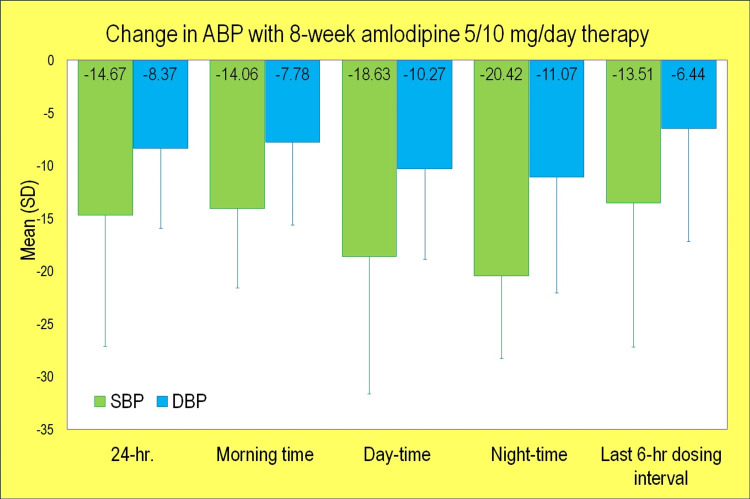
Change in ABP with 8-week amlodipine 5/10 mg/day therapy in PP dataset (n=49) ABP: Ambulatory blood pressure (mmHg); DBP: Diastolic blood pressure (mmHg); PP: Per-protocol; SBP: Systolic blood pressure (mmHg); SD: Standard deviation

Figure 1 presents ambulatory values (unadjusted) of SBP and DBP, respectively, for change from baseline in PP dataset (n=49).

Reduction in SBP and DBP in sub-groups

Table 4 presents the ambulatory SBP values for baseline, end of study and change from baseline in SBP in different sub-groups in PP dataset (n=49). Significant reductions in SBP (p<0.0001) measurements (office BP and ambulatory BP) were observed from baseline in both stage-1 and stage-2 hypertensive patients. Also, significant (p<0.0001) reductions in SBP measurements were observed in sub-groups based on treatment duration, amlodipine dose, and hypertension duration. No differences were observed between different genders and hypertension severity.

**Table 4:** Ambulatory 24-h SBP (mmHg) in different sub-groups in PP dataset (n=49) BMI: Body mass index; PP: Per protocol; SBP: Systolic blood pressure; SD: Standard deviation

			Baseline	8 weeks	Change	t-test	Effect size
	Sub-group	N	Mean (SD)	Mean (SD)	Mean (SD)	P	Cohen’s ‘d’
SBP (mm Hg)							
Occupation	Service	12	143.3 (7.7)	127.7 (5.0)	-15.00 (9.71)	<0.0001	1.95
	Self-employed / business	10	141.1 (7.1)	122.0 (6.2)	-19.13 (8.95)	<0.0001	2.69
	Unemployed	18	146.1 (10.8)	127.3 (11.4)	-17.43 (15.41)	<0.0001	1.61
	Retired	9	139.2 (7.8)	127.1 (10.7)	-13.87 (10.97)	<0.0001	1.77
Physical activity in life	Sedentary	23	141.1 (6.9)	129.3 (8.8)	-11.44 (10.49)	<0.0001	1.67
	Mild-to-moderate activity	13	141.5 (9.4)	126.3 (10.0)	-13.79 (9.19)	<0.0001	1.47
	Heavy physical activity	13	146.7 (11.1)	121.5 (7.7)	-26.55 (10.75)	<0.0001	2.39
Gender	Male	30	141.3 (7.7)	124.6 (8.2)	-16.83 (9.34)	<0.0001	2.18
	Female	19	146.1 (11.1)	129.0 (10.5)	-16.31 (16.19)	<0.0001	1.47
Amlodipine daily dose	Amlodipine 5 mg/d	28	138.2 (7.6)	124.1 (9.4)	-14.48 (11.79)	<0.0001	1.90
	Amlodipine 10 mg/d	21	147.3 (8.4)	128.5 (8.7)	-19.06 (12.19)	<0.0001	2.26
Duration of hypertension	<5 years	25	143.7 (9.9)	125.8 (8.8)	-17.48 (11.41)	<0.0001	1.77
	>= 5 years	24	141.6 (7.9)	126.8 (10.2)	-15.16 (13.42)	<0.0001	1.91
BMI category (WHO criteria)	Normal weight	6	142.0 (11.5)	125.0 (11.5)	-18.28 (11.13)	<0.0001	1.59
	Overweight	21	143.9 (6.9)	127.6 (8.5)	-15.73 (10.83)	<0.0001	2.27
	Obese	22	142.2 (10.9)	125.2 (9.7)	-17.06 (13.76)	<0.0001	1.56
Age group	<50 years	18	144.5 (11.1)	127.0 (5.9)	-16.36 (11.07)	<0.0001	1.47
	>= 50 years	31	142.2 (8.1)	125.6 (10.9)	-16.82 (12.86)	<0.0001	2.08
Hypertension severity	Stage-1 hypertension	34	138.3 (10.9)	124.9 (9.7)	-13.41 (11.85)	<0.0001	1.24
	Stage-2 hypertension	15	141.5 (17.4)	124.0 (9.3)	-17.53 (13.68)	<0.0001	1.01
Amlodipine therapy	Monotherapy	17	142.59 (13.29)	124.38 (11.72)	-18.21 (14.73)	<0.0001	1.23
	Combination therapy	32	137.56 (12.82)	124.28 (12.48)	-13.28 (14.79)	<0.0001	0.898

Table 5 presents the ambulatory DBP values for baseline, end of study and change from baseline in DBP in different sub-groups in PP dataset (n=49). Significant reductions in DBP (p, 0.001) measurements (office BP and ambulatory BP) were observed from baseline in both stage-1 and stage-2 hypertensive patients. Also, significant (p<0.0001) reductions in DBP measurements were observed in sub-groups based on treatment duration, amlodipine dose, and hypertension duration. No differences were observed between different genders and hypertension severity.

**Table 5:** Ambulatory 24-hr. DBP (mm Hg) in different sub-groups in PP dataset (n=49) BMI: Body mass index; DBP: Diastolic blood pressure; PP: Per protocol; SD: Standard deviation

			Baseline	8 weeks	Change	t-test	Effect size
	Sub-group	N	Mean (SD)	Mean (SD)	Mean (SD)	P	Cohen’s ‘d’
DBP (mm Hg)							
Occupation	Service	12	90.1 (6.4)	79.0 (3.4)	-10.86 (4.41)	<0.0001	1.68
	Self-employed / business	10	89.1 (3.6)	77.0 (4.6)	-12.00 (6.52)	<0.0001	3.37
	Unemployed	18	85.5 (12.8)	76.3 (10.1)	-8.36 (9.64)	<0.0001	0.65
	Retired	9	78.0 (7.3)	70.7 (4.7)	-6.73 (6.48)	<0.0001	0.92
Physical activity in life	Sedentary	23	82.9 (8.9)	76.0 (8.3)	-6.00 (6.07)	<0.0001	0.67
	Mild-to-moderate activity	13	83.0 (10.5)	74.8 (7.4)	-7.01 (6.15)	<0.0001	0.67
	Heavy physical activity	13	89.8 (10.5)	76.6 (6.6)	-16.09 (6.53)	<0.0001	1.53
Gender	Male	30	85.3 (8.0)	75.5 (5.6)	-10.05 (5.87)	<0.0001	1.25
	Female	19	84.7 (13.7)	76.5 (10.1)	-8.08 (10.08)	<0.0001	0.59
Amlodipine daily dose	Amlodipine 5 mg/day	28	81.1 (8.9)	72.8 (6.4)	-8.37 (7.23)	<0.0001	0.94
	Amlodipine 10 mg/day	21	88.8 (9.9)	79.3 (7.2)	-10.41 (8.03)	<0.0001	1.05
Duration of hypertension	<5 years	25	86.6 (10.9)	76.8 (7.8)	-9.70 (7.54)	<0.0001	0.89
	>= 5 years	24	82.5 (8.3)	74.2 (6.7)	-8.70 (7.91)	<0.0001	1.05
BMI category (WHO criteria)	Normal weight	6	81.8 (10.8)	75.5 (1.7)	-9.03 (7.96)	<0.0001	0.89
	Overweight	21	85.9 (8.9)	76.9 (8.6)	-9.00 (7.41)	<0.0001	1.05
	Obese	22	85.2 (11.5)	75.1 (7.3)	-9.71 (8.09)	<0.0001	0.83
Age group	<50 years	18	91.8 (7.7)	80.2 (4.7)	-10.86 (6.26)	<0.0001	1.02
	>= 50 years	31	81.7 (9.6)	73.1 (7.6)	-8.37 (8.30)	<0.0001	0.84
Hypertension severity	Stage-1 hypertension	34	81.6 (11.3)	73.7 (9.1)	-7.94 (7.09)	<0.0001	0.70
	Stage-2 hypertension	15	83.5 (13.4)	74.1 (7.4)	-9.33 (8.70)	0.001	0.70
Amlodipine therapy	Monotherapy	17	88.35 (11.48)	80.02 (10.36)	-8.33 (11.42)	0.008	0.73
	Combination therapy	32	78.91 (10.89)	73.46 (9.80)	-5.44 (9.34)	0.002	0.583

For all other parameters, no significant differences were observed for change in ambulatory BP with respect to age, hypertension duration, and baseline BMI. Greater reduction (p<0.05) in ambulatory BP were observed for all parameters except night-time SBP (p=0.061) were observed in patients treated for shorter period.

Dippers and non-dippers

At baseline, there were 13 (26.5%) dippers which increased to 28 (57.1%) at end of study. At baseline, there were 30 (61.2%) non- dippers which decreased to 18 (36.7%) at eight weeks. Thus, 40.0% non-dippers were converted to dippers with amlodipine therapy. However, these changes were not significant (c2=0.225, p=0.635, McNemar’s test).

Multivariate analyses for change in ambulatory BP

Results of multivariate analyses showed no significant effect of any of the independent variables (sub-groups) on the ambulatory BP (p>0.05).

Safety

No adverse effects were reported by any of the patients during the study period. Since the laboratory data was not available/not done for most of the patients at baseline and during follow-up visit, no comparisons were made.

## Discussion

The use of 24-h ABPM has improved the monitoring of antihypertensive treatment effectiveness throughout the dosing interval [4]. It helps the clinician decide on the optimum drug and dosing schedule for the treatment of hypertension. This post-marketing observational study captured the real-world BP control with eight-week amlodipine 5/10 mg/day therapy in patients with mild-to-moderate hypertension.

In a study that evaluated the association between morning surge in BP (based on ABPM) and cardiovascular events, a single daily dose of amlodipine has been reported to maintain BP control throughout 24 hours with minimal fluctuations [10]. This was attributed to the long half-life of amlodipine. We observed significant reductions (p<0.0001) from baseline in all BP parameters on ABPM at the end of the eight-week treatment with amlodipine. Similar improvements were observed in studies with amlodipine/olmesartan combination therapy by Parati et al. for improvements in 24-h BP control [11]. In another double-blind, randomized controlled trial conducted by Volpe et al., eight-week therapy with amlodipine/olmesartan (10/5, 20/5 was associated with significantly greater 24-h, daytime and night-time ambulatory BP reductions compared with monotherapy) [12]. A randomized controlled (RCT) study by Peng et al. comparing amlodipine and telmisartan as combination therapies at different times of the day (morning and bedtime) reported effective BP control irrespective of the time of administration [13]. Amlodipine has been found to show a similar reduction in BP compared to most of the commonly used drugs like angiotensin-converting enzyme inhibitors (ACE-Is), angiotensin receptor blockers (ARBs), beta-adrenergic receptor blockers, and other CCBs [9,14-17]. In a double-blind RCT by Coca et al. daily single dosage of amlodipine showed greater BP reduction compared to nitrendipine for its 24-h BP control [18]. In an 18-week, prospective, randomized, double-blind study from Brazil by Miranda et al., the amlodipine/ramipril FDC was associated with significantly reduced ambulatory and office-measured BP compared with amlodipine monotherapy, with the exception of office DBP [19].

An association between blunted sleep-time BP decline (non-dipping) and increased cardiovascular disease (CVD) risk in hypertension has been well documented in the past [20-22]. A meta-analysis of 17,312 hypertensive patients reports a 27% higher risk for total cardiovascular events (CVEs) in non-dippers compared to dippers [23]. We observed a marginal but non-significant (p>0.05) increase in the proportion of dippers and extreme dippers with amlodipine. Amlodipine reduces the night-time BP both in the non-dippers and dippers and significant positive correlations are found between baseline BP levels and the BP reduction after amlodipine therapy [24]. There is a trend for better BP lowering and less BP variability when the medications are administered at bedtime [13]. Similarly, a study in 98 non-dipper hypertensive patients show that night-time dosing of amlodipine demonstrated effects like day-time administration [25]. The time of administration of amlodipine has no effect on its antihypertensive efficacy [26]. Our results of multivariate analyses for change in ambulatory BP showed no significant (p>0.05) effect of factors like gender, the severity of hypertension, the daily dose of amlodipine, duration of hypertension, BMI category, age group, and duration of amlodipine therapy. This suggests that amlodipine therapy can be useful as monotherapy or combination therapy in all types of patients irrespective of gender, severity of hypertension, duration of hypertension, BMI, and age.

We did not observe any adverse effects with amlodipine-based therapy over an eight-week period. However, in the RCT from Brazil, Miranda et al. reported that 30.6% (41/105) of patients treated with amlodipine monotherapy experienced adverse events which were considered possibly related to the study drug [15]. This study has some limitations in terms of being a post-marketing study and a small sample size. Due to the observational, non-comparative study design, strict control of confounding factors could not be obtained. Hence, this limits the extrapolation of study results to large-scale population.

## Conclusions

Amlodipine 5/10 mg/day therapy used as monotherapy or adjuvant therapy provides significant reductions in both office BP and ambulatory BP over 8 weeks. BP reductions were seen by 14.2 mmHg in SBP and 13.6 mmHg in DBP with 10 mg Amlodipine at the end of eight weeks, and the non-dipper-to-dipper conversion was 40.0%. Being devoid of potential adverse effects, such as reflex tachycardia and first dose effect, amlodipine can be one of the safe alternatives as monotherapy or add-on therapy in the management of hypertension.
